# The Influence of an Evidence-Based Practice Course on Saudi Nursing Students’ Beliefs About and Implementation of Evidence-Based Practice: A Cross-Sectional Comparative Study

**DOI:** 10.7759/cureus.72243

**Published:** 2024-10-23

**Authors:** Manal H Abo Elmagd, Muna Alharbi, Wael Alhazmi

**Affiliations:** 1 Community Nursing and Healthcare, Umm Al-Qura University, Mecca, SAU

**Keywords:** evidence-based practice beliefs, evidence-based practice course, evidence-based practice implementation, nursing education, nursing student.

## Abstract

Background

Previous research on evidence-based practice (EBP) beliefs and implementation among nurses in daily clinical practice has revealed a strong belief in EBP, yet its implementation remains inadequate. To prepare prospective nurses to meet this requirement, academic efforts have been made to incorporate EBP into the undergraduate nursing curriculum.

Objective

This study aims to examine the influence of a course focused on EBP on Saudi nursing students’ beliefs about and implementation of EBP at one university using a cross-sectional comparative design.

Method

A cross-sectional comparative design was employed. The Evidence-Based Practice Belief Scale and the Evidence-Based Practice Implementation Scale were utilized. A questionnaire was distributed to 243 second-year undergraduate nursing students before they began the EBP course and after they completed it.

Result

A total of 130 students responded to the questionnaire before they started the course and 118 students after they completed the course. The results show that the mean score on the EBP Belief Scale (EBPB) was 55.69 ± 9.93 before the course but increased to 60.75 ± 11.45 afterward. Similarly, the mean score on the Evidence-Based Practice Implementation Scale (EBPI) was 31.59 ± 18.39 prior to the course and increased to 46.41 ± 21.93 post-course.

Conclusion

These findings suggest that students internalized the importance of staying updated on the latest research and expressed a willingness to implement EBP across advanced healthcare systems, which could contribute to positive patient outcomes. Additional practical workshops and training programs are essential to equip nurses with the knowledge and skills needed to enhance safe nursing care in clinical settings.

## Introduction

Quality nursing care is expected by all patients and consumers of health services. Adherence by nurses to evidence-based practice (EPB) is essential for achieving high-quality nursing care [1]. EBP is defined as utilizing the best available research evidence to guide clinical practice, ensuring patients receive the best possible nursing care [2].

Prioritizing EBP in nursing education is crucial, as it serves as a foundation for healthcare professionalism. Currently, it's widely recognized that nurses must be proficient in EBP to meet the demands of modern healthcare. To facilitate this, EBP courses are integrated into undergraduate nursing programs. The primary goal is to equip future nurses with the skills and knowledge required to effectively implement EBP in their practice. This integration enables the nurse to critically evaluate evidence, incorporate it into their clinical decision-making process, and continually assess the outcomes of their interventions. By embedding EBP into undergraduate nursing education, healthcare organizations can ensure that their workforce is well-prepared to deliver high-quality, evidence-based care to patients [3-4].

Many research studies emphasize that EBP is not just an important competency that healthcare providers acquire after employment, but should also be introduced during their undergraduate studies. This can be achieved by enhancing students’ knowledge, skills, and attitudes toward EBP [4]. Nursing students need instructions not only in recognizing the importance of EBP but also in mastering the skills to locate, evaluate, and appropriately apply evidence when necessary. This approach will expand nursing knowledge, improve the practices of aspiring nurses, and enhance patient outcomes [5].

Strengthening nurses' beliefs about EBP has been shown to facilitate its implementation. These beliefs encompass nurses’ opinions on the value and benefits of EBP, as well as their confidence in their own EBP expertise [6]. Nurses who strongly believe in EBP are more likely to utilize it than those with weaker beliefs [7].

The implementation of evidence-based practice (IEBP) refers to the application of the most current, credible, and relevant research findings alongside practical experience and established standards for clinical decision-making [8]. However, previous research on IEBP by nurses in their daily clinical practice has shown that it is often inadequate [6].

A study conducted at a nursing faculty in Saudi Arabia reported that students expressed a strong belief in EBP but demonstrated low levels of its implementation [3]. These findings are consistent with a previous study conducted in Portugal, where both, educators and nursing students reported strong beliefs in EBP alongside low levels of its implementation [9]. Therefore, it is suggested that strategies be tested in educational contexts to improve the implementation of EBP [3].

The literature lacks clarity on how implementing a specific EBP course in Saudi universities influences students’ beliefs and practices regarding EBP. Quality improvement initiatives could play a vital role in enhancing EBP implementation. By incorporating an EBP-focused curriculum, students can engage in practical applications of EBP, allowing them to experience firsthand the impact of evidence-based interventions on patient care and outcomes. Incorporating these strategies not only enhances the educational experience for nursing students but also promotes a culture of EBP within healthcare settings. This study extends previous research that examined students' beliefs and willingness to implement EBP before enrolling in the course, using that study's data as baseline information [10]. The current study aims to investigate the influence of an EBP course on Saudi nursing students’ beliefs about and implementation of EBP at a particular university, utilizing a cross-sectional comparative design.

## Materials and methods

Design

This study employed a cross-sectional comparative design, which is suitable for comparing data collected before and after the evidence-based practice course (EBPC) at a single point in time.

Participants and setting

In the Faculty of Nursing at Umm Al-Qura University, the standard study plan requires students to take an EBPC in their second year of study. The EBPC aims to introduce nursing students to the fundamental knowledge and skills related to EBP concepts in nursing. It emphasizes the EBP process and the application of current, high-quality research evidence in clinical and healthcare settings. The course is structured around the seven steps of EBP: step 0: cultivate a spirit of inquiry within an EBP culture and environment; step 1: ask the burning clinical question in PICOT, i.e. patient, intervention, comparison, outcome and (sometimes) time format; step 2: search for and collect the most relevant evidence; step 3: critically appraise the evidence; step 4: integrate the best evidence with clinical expertise and patient/family preferences in making practice decisions or changes; step 5: evaluate outcomes of the practice decisions or changes; and step 6: disseminate the outcomes of the EBP decisions or changes [6].

The inclusion criteria were that the participants must be at least 18 years old, must be enrolled in the nursing faculty at Umm Al-Qura University, and must be eligible to undertake the EBPC and successfully complete it. Students not enrolled in the nursing faculty at Umm Al-Qura University; students who have not completed the prerequisite courses for enrolling in the EBPC, and students who do not complete the EBPC were excluded from the study/

The total number of the student population who met the criteria of the study was 163 female and 80 male students in the second year (n=243). A convenience sample of this population responded to the voluntarily self-reported questionnaire. This questionnaire was written in English and distributed to the students before they began the EBPC, and again after completing the course. The course ran from April to June 2023, with pre-test data collected from February 2023 and post-test data collected until September 2023.

Data collection tool

The Evidence-Based Practice Belief Scale and the Evidence-Based Practice Implementation Scale were used [11-12]. Both scales have been used among nurses to test their reliability and validity [12]. Both scales exhibited strong reliability, with Cronbach's alpha values greater than 0.90 for each scale, suggesting excellent internal consistency. Additionally, principal component analysis verified that each scale assesses a single, unidimensional construct, further supporting their validity [12]. Three additional questions were added: Do you attend any programs related to EBP? Do you know the meaning of EBP? and Are you willing to apply nursing care informed by EBP? In addition, demographic data on age and sex were collected. The survey was disseminated via Qualtrics and distributed to students via email and various social media platforms.

The EBPB Scale consists of sixteen statements that assess the self-reported beliefs of participants regarding the importance of EBP and their confidence in their knowledge and skills related to EBP [12]. Participants are asked to indicate the level to which they agree or disagree with the sixteen statements by answering on a 5-point Likert scale ranging from “strongly disagree” (1) to “strongly agree” (5). The scores are added up to a minimum of sixteen points and a maximum of eighty. Higher scores reflect more positive beliefs about EBP.

To study the nurses’ beliefs about EBP, we used four subscales in the analysis of the EBP Beliefs Scale, defined as (1) knowledge beliefs, (2) value beliefs, (3) resource beliefs, and (4) time and difficulty beliefs [13]. The items related to knowledge beliefs touch on knowing the steps of EBP, measuring outcomes, implementing practice changes, and confidence in the ability to implement EBP (n = 5). The value items focus on beliefs about how EBP leads to optimal clinical care and enhances patient outcomes (5 items). The resource items assess access to top-notch resources and the ability to overcome obstacles (4 items). Time and difficulty beliefs encompass questions about the time investment needed for EBP implementation and nurses' perceptions of its difficulty (2 items).

The EBP Implementation Scale comprises eighteen statements, and participants rate their frequency of agreement using a 5-point scale [12]. The questions are related to the utilization of evidence-based practice in professional activity and assess the fundamental elements and steps of EBP. For example, they ask how long the respondent has “Evaluated the outcomes of a practice change” or “Used an EBP guideline or systematic review to change clinical practice”, and responses are scored on a 5-point Likert scale that ranges from “not at all” (1) to “to a great extent” (5). Scores are calculated by summing the responses to the eighteen items, for a total score that can range from eighteen to ninety. Higher total scores reflect more frequent use of EBP [12].

Data analysis

Version 24 of the Statistical Package for Social Sciences (SPSS) was used to analyze the data in this study. The statistical tests employed included both descriptive and inferential statistics: descriptive statistics comprised frequencies, means, and standard deviations, while inferential statistics included t-tests, chi-square for independence, and pearson correlation coefficient to examine correlations between study variables.

Ethical considerations

This study received ethical approval from the Umm Al-Qura University Ethics Committee (Approval No. HAPO-02-K-012-2023-02-1444). The first page of the questionnaire included an explanatory statement outlining the study’s purpose and methods, as well as information on how participants’ anonymity would be maintained. It also identified the benefits and risks associated with the study. Aside from the expected time commitment required to complete the questionnaire, there were no significant risks involved in this research. The students had the right to withdraw from the survey at any time before submission, as the data were anonymous and not linked to identifiable information.

## Results

The number of participants in pre-EBPC was n=130 out of 243, resulting in a response rate of 53.5%. In post-EBPC, the number of participants was n=118 out of 243, yielding a response rate of 48.5%. Table 1 shows that the mean age of the students was 20 ± 0.08 years, while 50 (pre-test) and 59 (post-test) of the students were male. The table highlights that 65.38% and 97.5% of the students knew the meaning of EBP before and after taking the course, respectively. It was observed that 68.46% of the students intended to implement EBP in nursing care before the course, and 87.3% did so afterwards.

**Table 1:** Demographic data of the participants EBPC: evidence-based practice course

		pre-EBPC (n = 130)		post-EBPC (n = 118)	
Variable		No.	%	No.	%
Age	21-22years	25	19.23%	21	17.79%
	19-20years	105	80.77%	97	82.21%
Mean age ± SD		20 ± 0.08			
Gender	Male	50	38.46%	59	50%
	Female	80	61.54%	59	50%
Do you attend any programs related to evidence-based practice?	Yes	37	28.46%	109	92.4%
	No	93	71.54%	9	7.60%
Do you know the meaning of “evidence-based practice”?	Yes	85	65.38%	115	97.5%
	No	45	34.62%	3	2.50%
Are you willing to apply evidence-based practice in nursing care?	Yes	89	68.46%	103	87.3%
	No	41	31.54%	15	12.70%

Regarding knowledge beliefs, Table 2 shows that 60.77% and 72.03% of the students agree and strongly agree that they were sure enough that they could implement EBP, before and after taking the EBPC respectively. Additionally, 50% and 83.05% strongly agreed/agreed that the steps of EBP were clear enough for them pre- and post-EBPC, respectively; 49.23 % and 76.27% strongly agreed that they knew how to implement EBP well enough to make practice changes. Regarding EBP value beliefs, 70% and 86.44% of students pre- and post-EBPC, respectively, reported a strong belief that EBP results in the best clinical care for patients; 67.69% and 85.59% agreed and strongly agreed that they were sure evidence-based guidelines can improve clinical care.

**Table 2:** Distribution of EBPB belief subscale scores among students before and after an evidence-based practice course (EBPC) X^2^; Chi-square for independence, EBP: evidence-based practice

EBPB subscale	pre-EBPC (n = 130)				post-EBPC (n = 118)			X^2^	p- value	
	Strongly Agree /Agree	Neither agree nor disagree		Disagree Strongly disagree	Strongly Agree /Agree	Neither agree nor disagree	Disagree Strongly disagree			
	No. %	No. %		No. %	No. %	No. %	No. %			
Knowledge beliefs										
2-I am clear about the steps of EBP	65 (50.00)	23 (17.69)	42 (32.31)		98 (83.05)	8 (6.78)	12 (10.17)	11.0	0.19	
3-I am sure that I can implement EBP	79 (60.77)	33 (25.38)	18 (13.85)		85 (72.03)	20 (16.95)	13 (11.02)	13.6	0.60	
10-I am sure about how to measure the outcomes of clinical care	70 (53.85)	36 (27.69)	24 (18.46)		86 (72.88)	17 (14.41)	15 (12.71)	6.48	0.59	
14-I know how to implement EBP well enough to make practice changes.	64 (49.23)	42 (32.31)	24 (18.46)		90 (76.27)	16 (13.56)	12 (10.17)	1.57	0.81	
15- I am confident about my ability to improve EBP where I work.	77 (59.23)	36 (27.69)	17 (13.08)		91 (77.12)	16 (13.56)	11 (9.32)	19.55	0.24	
Value beliefs										
1-I believe that EBP results in the best clinical care for patients	91 (70.00)	30 (23.08)	9 (6.92)		102 (86.44)	10 (8.47)	6 (5.08)	8.11		0.42
4-I believe that critically appraising evidence is an important step in the EBP process	85 (65.38)	32 (24.62)	13 (10)		96 (81.36)	14 (11.86)	8 (6.78)	24.0		0.02*
5-I am sure that evidence-based guidelines can improve clinical care	88 (67.69)	27 (20.77)	15 (11.54)		101 (85.59)	8 (6.78)	9 (7.63)	8.92		0.34
9-I am sure that implementing EBP will improve the care that I deliver to my patients	90 (69.23)	29 (22.31)	11 (8.46)		95 (80.51)	12 (10.17)	11 (9.32)	8.88		0.05*
16-I believe the care that I deliver is evidence-based	75 (57.69)	45 (34.62)	10 (7.69)		92 (77.97)	14 (11.86)	12 (10.17)	1.24		0.87
Resource access										
6-I believe that I can search for the best evidence to answer clinical questions in a time-efficient way	75 (57.69)	38 (29.23)	17 (13.08)		90 (76.27)	15 (12.71)	13 (11.02)	11.59		0.07
7-I believe that I can overcome barriers in implementing EBP	67 (51.54)	48 (36.92)	15 (11.54)		89 (75.42)	18 (15.25)	11 (9.32)	8.44		0.07
8-I am sure that I can implement EBP in a time-efficient way	66 (50.77)	48 (36.92)	16 (12.31)		83 (70.34)	18 (15.25)	17 (14.41)	9.33		0.15
12-I am sure that I can access the best resources in order to implement EBP	71 (54.62)	40 (30.77)	19 (14.62)		91 (77.12)	20 (16.95)	7 (5.93)	3.36		0.67
Time difficulties										
11-I believe that EBP takes too much time	14 (10.77)	49 (37.69)	67 (51.54)		15 (12.71)	11 (9.32)	92 (77.97)	6.53		0.58
13-I believe EBP is difficult	22 (16.92)	51 (39.23)	57 (43.85)		13 (11.02)	16 (13.56)	89 (75.42)	5.28		0.21

Concerning resource access, 57.69% and 76.27% of students pre- and post-EBPC, respectively, agreed and strongly agreed that they can search for the best evidence to answer clinical questions in a time-efficient way; 51.54% and 75.42% strongly agreed that they can overcome barriers to implementing EBP. Finally, regarding time difficulties, 10.77% (before the EBPC) and only 12.71% (after the EBPC) of students agreed and strongly believed that EBP adherence takes too much time. A total of 16.92% and 11.02% of students before and after EBPC, respectively, agreed and strongly agreed that EBP is difficult.

Table 3 shows that 30.77% and 64.41% of the students generated a PICO (patient/population, intervention, comparison and outcomes) question before and after attending the EBPC, respectively. Of the students, 29.23% and 52.54% reported that they were able to critically appraise evidence from a research study. Promisingly, 40% and 63.56% of the students reported being able to evaluate the outcomes of a practice change pre- and post-EBPC, respectively. Meanwhile, 22.31% and 55.08% of the students accessed the National Guidelines Clearinghouse, and 23.08% and 56.78% accessed the Cochrane Database of Systematic Reviews pre- and post-EBPC, respectively. Of the students, 33.08% and 61.86% promoted the use of EBP to their colleagues pre- and post-EBPC attendance. In addition, 36.92% and 58.47% of the students reported they had shared outcome data they collected with their colleagues pre- and post-EBPC, respectively. 

**Table 3:** Distribution of participants' responses to EBPI scale before and after EBPC attendance X^2^; Chi-square for independance, EBPC:evidence-based practice course; PICO: patient/population, intervention, comparison and outcomes; EBP: evidence-based practice

EBPB subscale	pre-EBPC (n = 130)			post-EBPC (n = 118)			X^2^	p- value
	Not at all	To small/ Some extent	To moderate /a great extent	Not at all	To small/ some extent	To moderate/a great extent		
	No. (%)	No. (%)	No. (%)	No. (%)	No. (%)	No. (%)		
1. Used evidence to change my clinical practice	24 (18.46)	59 (45.38)	47 (36.15)	17 (14.41)	37 (31.36)	64 (54.24)	1.90	0.92
2. Critically appraised evidence from a research study	24 (18.46)	68 (52.31)	38 (29.23)	12 (10.17)	44 (37.29)	62 (52.54)	5.39	0.24
3. Generated a PICO question about my clinical practice	25 (19.23)	65 (50)	40 (30.77)	10 (8.47)	32 (27.12)	76 (64.41)	3.93	0.41
4. Informally discussed evidence from a research study with a colleague	22 (16.92)	66 (50.77)	42 (32.31)	9 (7.63)	33 (27.97)	68 (57.63)	2.56	0.63
5. Collected data on a patient problem	22 (16.92)	61 (46.92)	47 (36.17)	11 (9.32)	27 (22.88)	80 (67.80)	5.61	0.23
6. Shared evidence from a study/studies in the form of a report or presentation to > 2 colleagues.	28 (21.54)	66 (50.77)	36 (27.69)	15 (12.71)	36 (30.51)	67 (56.78)	4.41	0.35
7. Evaluated the outcomes of a practice change	23 (17.69)	55 (42.31)	52 (40)	12 (10.17)	31 (26.27)	75 (63.56)	1.37	0.84
8. Shared an EBP guideline with a colleague	31 (23.85)	63 (48.46)	36 (27.69)	12 (10.17)	32 (27.12)	74 (62.71)	2.93	0.57
9. Shared evidence from a research study with a patient/family member	28 (21.54)	62 (47.69)	40 (30.77)	13 (11.02)	30 (25.42)	75 (63.56)	5.44	0.24
10. Shared evidence from a research study with a multidisciplinary team	30 (23.08)	63 (48.46)	37 (28.46)	15 (12.71)	29 (24.58)	74 (62.71)	1.49	0.82
11. Read and critically appraised a clinical research study	25 (19.23)	58 (44.62)	47 (36.15)	13 (11.02)	33 (27.97)	72 (61.02)	3.33	0.50
12. Accessed the Cochrane Database of Systematic Reviews	28 (21.54)	72 (55.38)	30 (23.08)	13 (11.02)	38 (32.20)	67 (56.78)	4.77	0.31
13. Accessed the National Guidelines Clearinghouse	31 (23.85)	70 (53.85)	29 (22.31)	15 (12.71)	38 (32.20)	65 (55.08)	7.73	0.10
14. Used an EBP guideline or systematic review to change clinical practice	27 (20.77)	55 (42.31)	48 (36.92)	16 (13.56)	29 (24.58)	73 (61.86)	4.54	0.32
15. Evaluated a care initiative by collecting patient outcome data	27 (20.77)	56 (43.08)	47 (36.15)	18 (15.25)	31 (26.27)	69 (58.47)	5.06	0.28
16-Shared the outcome data collected with colleagues	27 (20.77)	55 (42.31)	48 (36.92)	14 (11.86)	35 (29.66)	69 (58.47)	0.74	0.92
17-Changed practices based on patient outcome data	26 (20.00)	60 (46.15)	44 (33.85)	16 (13.56)	35 (29.66)	67 (56.78)	3.42	0.48
18-Promoted the use of EBP to my colleagues	33 (25.38)	54 (41.54)	43 (33.08)	10 (8.47)	35 (29.66)	73 (61.86)	10.18	0.03*

Table 4 shows that the mean score on the EBPB scale was 55.69 ± 9.93 before the EBPC but increased to 60.75 ± 11.45 afterward. There is a highly statistically significant difference between the students’ mean scores on the EBPB scale pre- and post-EBPC attendance (t-test value = -3.527; p-value = <0.001**). In addition, the mean EBPI scale score was 31.59 ± 18.39 before EBPC attendance but increased to 46.41 ± 21.93 after the EBPC. A highly statistically significant difference was found between students’ mean scores on the EBPI scale pre- and post-EBPC (t-test value: -5.641; p-value: <0.001**).

**Table 4:** A comparison between the nursing students’ mean EBPB and EBPI scores pre- and post-EBPC t; t-test

Study variables	pre-EBPC (n = 130)		post-EBPC (n = 118)		t-test	df	effect size	p-value
	Mean	SD	Mean	SD				
Evidence-Based Beliefs Scale	55.69	9.93	60.75	11.45	- 3.527	117	0.09	<0.001**
Evidence-Based Implementation Scale	31.59	18.39	46.41	21.93	- 5.641	117	0.21	<0.001**

As Table 5 shows that, based on the Pearson correlation test, there was a strong, significant positive correlation between nursing students’ EBPB and EBPI scores after EBPC attendance (R-value: 0.544; P-value: <0.001). Meanwhile, there was a weak and insignificant correlation between nursing students’ EBPB and EBPI scores before the EBPC (R-value: 0.154; P-value: 0.08).

**Table 5:** Coefficient correlation between nursing students’ EBPB and EBPI scores pre- and post-EBPC attendance R: Pearson correlation coefficient

Variable tested		Evidence-Based Implementation Scale (pre-EBPC)	Evidence-Based Implementation Scale (post-EBPC)
Evidence-Based Beliefs Scale (pre-EBPC)	R-Value	0.154	-
	P-Value	0.080	-
	N	130	-
Evidence-Based Beliefs Scale (post-EBPC)	R-Value	-	0.544
	P-value	-	<0.001**
	N	-	118

## Discussion

This study aims to examine the influence of an EBPC on Saudi nursing students’ beliefs about and implementation of EBP. The results indicate improvements in all items of the EBP Belief and Implementation scales from before to after the course. These enhancements are attributed to the educational course provided by the faculty of nursing to the participating students. This finding aligns with previous research showing that undergraduate nursing students have a very positive attitude toward EBP and its importance in delivering quality patient care; over 70% believed that EBP leads to high-quality clinical care and that evidence-based guidelines can enhance clinical practice [5].

Other researchers have reported similar outcomes, noting that participants in their studies valued EBP highly, as evidenced by high scores on the EBP belief scale [3]. This suggests that respondents perceive EBP as significant in nursing practice, recognizing its potential to improve the quality of care provided to patients.

Regarding the resource access subscale, undergraduate nursing students provided positive responses for all items both before and after the course implementation. Furthermore, most participants expressed a willingness to apply EBP in nursing care. This interest not only motivates them to learn about EBP but also drives them to overcome potential barriers to its application. Although the scores on the resource access subscale were positive before the course, they increased even further afterward.

Another interesting finding of this study is that, during the pretest period, a significant percentage of students believed that EBP is difficult and time-consuming. However, this perception improved in the post-test period as a result of the EBPC. This finding contradicts a previous study that reported most respondents agreed that EBP is difficult and time-consuming. It is suggested that completing an EBP course helps students manage their time effectively when implementing EBP [3].

Regarding the total mean scores on the EBP belief and EBP implementation scales, there were statistically significant improvements in both scales from before to after the course. These findings align with existing literature, which found that nursing students’ overall post-test EBP scores were significantly higher than their pre-test scores [14]. Additionally, other studies reported that undergraduate nursing students participating in an EBP program incorporating blended learning techniques exhibited notable enhancements in self-efficacy, understanding of EBP, and utilization of evidence, compared to a control group, within a two-month period [15]. Furthermore, the findings of this study support the recommendations of previous research, which stated that an educational program on EBP can effectively enhance the knowledge and skills of undergraduate nursing students [9].

The statistical results of this study will provide valuable insights for improving the EBPC. It is essential for clinicians and aspiring nurses to incorporate EBP into their nursing programs and practices, as EBP is a cornerstone of nursing excellence. By grounding their practices in strong evidence, nurses can optimize patient care, enhance safety, and drive quality improvement.

EBP emphasizes the importance of a patient’s preferences and values. By involving patients in their own care and tailoring care plans to their unique circumstances, nurses can improve patient satisfaction and well-being. Additionally, practical workshops and training programs are vital for equipping nurses with the knowledge and skills necessary to implement EBP effectively in clinical settings.

These initiatives help nurses make informed decisions, standardize care procedures, and enhance the credibility of the profession. When nurses utilize research-based evidence to guide their decisions and practices, their investment of time and effort can lead to more positive patient outcomes.

Limitations

This study has limitations that should be taken into account. First, the research was conducted at a single university in western Saudi Arabia, which may limit the generalizability of the results to other settings. Additionally, the sample used was subject to selection bias, as participation was voluntary among second-year students. Finally, there were many instructors in this course which may affect the results of the students.

## Conclusions

In this study, significant improvements were observed in the average means of the EBP Belief and Implementation scales from the pre-course period to after the test. These enhancements can be attributed to the educational courses-specifically the EBP course provided by faculty members for the nursing students who participated in the study. The EBP teaching process integrated fundamental steps of evidence-based nursing practice with tutorial demonstration sessions on how to apply these steps, thereby enhancing future nurses' practice. Students learned the importance of evidence-based knowledge and how to access, appraise, and apply it effectively. It is recommended that students be encouraged to attend additional training and workshops to further strengthen their EBP knowledge and skills.

Furthermore, the results indicate a significant positive correlation between the nursing students’ EBPB and EBPI scores following the EBPC. These findings suggest that students internalized the importance of staying updated on the latest research and expressed a willingness to implement EBP in advanced healthcare systems, which could help ensure positive patient outcomes. It is recommended that future research conduct a longitudinal study to measure the long-term impacts of EBPC on nursing students' beliefs and implementations, including after they become registered nurses. Additionally, exploring students' beliefs and willingness to implement EBP through qualitative research would complement the quantitative results and enhance understanding of these experiences. Furthermore, it is suggested to examine the factors that influence the integration of the EBPC principles into the overall nursing curriculum.
